# Supplementary material for: A Likelihood Approach for Real-Time Calibration of Stochastic Compartmental Epidemic Models
Source: PLoS Comput Biol. 2017 Jan 17;13(1):e1005257. doi: 10.1371/journal.pcbi.1005257 (PMC5240920; doi:10.1371/journal.pcbi.1005257)
Supplement: S1 File — (TAR.GZ) [file pcbi.1005257.s014.tar.gz › HSPH_Online-SI-Revision/IPoi09/n1-mild/n1-mild_BENCH_summary.pdf]

peak -8	R0	$1/\theta_2$	inf	inflog	newly	newlylog	Reff	AttackRate
mean	33.2	50.5	181.7	0.6	208.7	0.6	37.3	65.9
median	21.	40.1	141.2	0.5	156.9	0.6	30.1	61.9
Quantile .1	10.5	24.	61.4	0.3	78.8	0.4	16.3	27.8
Quantile .9	69.2	107.2	333.7	0.8	413.8	0.8	67.7	112.8
26	33	34						
length	50							

peak -4	R0	$1/\theta_2$	inf	inflog	newly	newlylog	Reff	AttackRate
mean	31.5	48.7	173.9	0.5	137.7	0.5	30.2	61.8
median	31.5	38.5	138.3	0.4	104.5	0.4	28.2	63.5
Quantile .1	4.2	14.	60.1	0.3	61.6	0.2	17.2	27.2
Quantile .9	57.7	96.5	334.	0.7	251.8	0.6	40.9	100.7
8	20	13						
length	50							

peak	R0	$1/\theta_2$	inf	inflog	newly	newlylog	Reff	AttackRate
mean	49.7	69.4	206.6	0.5	115.7	0.3	30.7	82.5
median	48.1	34.1	171.5	0.5	93.5	0.3	28.7	78.4
Quantile .1	17.	12.2	73.2	0.3	39.4	0.2	21.	45.8
Quantile .9	83.2	180.6	330.	0.7	212.4	0.5	42.2	116.7
0	11	0						
length	50							

peak +4	R0	$1/\theta_2$	inf	inflog	newly	newlylog	Reff	AttackRate
mean	49.3	69.7	152 849.	0.5	915.9	0.4	34.6	83.1
median	51.9	46.1	202.2	0.5	120.1	0.3	33.3	82.
Quantile .1	24.6	13.4	60.9	0.3	58.9	0.2	24.3	46.2
Quantile .9	74.1	170.1	702.1	0.9	234.	0.7	46.3	114.2
0	4	0						
length	50							

peak +8	R0	$1/\theta_2$	inf	inflog	newly	newlylog	Reff	AttackRate
mean	49.	65.2	132 467.	0.6	1043.7	0.5	39.4	83.4
median	51.6	50.	207.2	0.5	123.4	0.4	40.	80.3
Quantile .1	23.7	10.1	73.3	0.3	64.4	0.2	27.9	49.4
Quantile .9	67.6	142.5	1958.1	1.1	1273.5	1.	51.4	115.9
0	5	0						
length	50							
